# Assessment of CD52 expression in "double-hit" and "double-expressor" lymphomas: Implications for clinical trial eligibility

**DOI:** 10.1371/journal.pone.0199708

**Published:** 2018-07-18

**Authors:** Jeffrey W. Craig, Michael J. Mina, Jennifer L. Crombie, Ann S. LaCasce, David M. Weinstock, Geraldine S. Pinkus, Olga Pozdnyakova

**Affiliations:** 1 Department of Pathology, Brigham and Women’s Hospital, Boston, Massachusetts, United States of America; 2 Harvard Medical School, Boston, Massachusetts, United States of America; 3 Department of Medical Oncology, Dana-Farber Cancer Institute, Boston, Massachusetts, United States of America; 4 Broad Institute of Harvard and Massachusetts Institute of Technology, Cambridge, Massachusetts, United States of America; European Institute of Oncology, ITALY

## Abstract

"Double-hit" and "double-expressor" lymphomas represent distinct but overlapping subsets of aggressive B-cell non-Hodgkin lymphoma. The high rates of bone marrow involvement by these lymphomas pose a major therapeutic challenge due to the chemotherapy-resistant nature of the bone marrow microenvironment and the limited utility of rituximab-based salvage regimens in patients with relapsed/refractory disease. Preclinical studies utilizing high-dose cyclophosphamide in combination with the anti-CD52 monoclonal antibody alemtuzumab have recently shown promise in the treatment of intramedullary disease, and a Phase I human trial is now underway. In support of such efforts, here we perform CD52 target validation on a series of double-hit (n = 40) and double-expressor (n = 58) lymphomas using immunohistochemistry. CD52 expression levels varied considerably across samples, however positive staining was observed in 75% of both double-hit and double-expressor lymphomas. Similarly, high levels of CD52 expression were seen in patients whose disease was associated with high-risk clinical features, including primary refractory status (73%), higher IPI score (76%), and bone marrow involvement (74%). CD52 expression was not significantly correlated with diagnostically relevant pathologic features such as morphology, cytogenetic findings or other immunophenotypic features, but was notably present in all cases lacking CD20 expression (n = 6). We propose that CD52 expression status be evaluated on a case-by-case basis to guide eligibility for clinical trial enrollment.

## Introduction

High-grade B-cell lymphomas (HGBCL) with *MYC* and *BCL2* and/or *BCL6* rearrangements (i.e. "double-hit" lymphomas; DHL; 6–9% of aggressive B-cell lymphomas) and the subset of diffuse large B-cell lymphomas (DLBCL) and HGBCL, not otherwise specified (NOS), with MYC and BCL2 protein over-expression (i.e. "double-expressor" lymphomas; DEL; 25–30% of remaining aggressive B-cell lymphomas) represent distinct but overlapping subsets of mature B-cell non-Hodgkin lymphomas with aggressive clinical course, poor response to conventional chemotherapy (i.e. R-CHOP) and high relapse rates [1, 2]. While the prognosis of DHL is worse than that of DEL, both show inferior overall and progression-free survival compared to non-double-expressor DLBCL, even after accounting for the presence of other high-risk features [3, 4]. These recently defined lymphoma categories represent major therapeutic challenges, in large part due to the high failure rates of initial and traditional salvage chemotherapy regimens in patients with relapsed/refractory disease.

Along with performance status at diagnosis, bone marrow (BM) involvement is considered one of the strongest prognostic findings in patients with DHL [5]. The negative impact of BM involvement has been attributed to the treatment-resistant nature of the BM microenvironment, which is capable of suppressing anti-tumor macrophage number and activation [6]. Given the primary role of macrophages in antibody-mediated antitumor activity in this context, novel treatment approaches that improve the efficacy of therapeutic antibodies through enhanced effector cell responses are considered highly desirable. To this end, work by Pallasch et al. has shown that the therapeutic antibody-refractory nature of the BM microenvironment can be temporarily abrogated through the synergistic effects of high-dose cyclophosphamide (CTX), which induces the release of stress-associated cytokines by leukemic cells, ultimately leading to macrophage recruitment and phagocytosis [7]. The potential of this therapeutic strategy has more recently been demonstrated in human-derived xenografts taken from patients with relapsed/refractory DHL [8].

These aforementioned studies utilized high-dose CTX in combination with the humanized IgG1 kappa monoclonal antibody alemtuzumab (Campath-1H), which works by targeting CD52, a GPI-linked glycoprotein that serves as a costimulatory molecule for the induction of T-regulatory cells and is highly expressed on essentially all B and T lymphocytes, the majority of monocytes, macrophages and NK cells, and a subpopulation of granulocytes [9, 10]. The *in vivo* cytolytic effects of alemtuzumab preferentially target lymphocytes of the adaptive immune system, while leaving innate immune cells relatively intact [11]. As a result, alemtuzumab has found use in the treatment of B-cell chronic lymphocytic leukemia (CLL) and T-cell prolymphocytic leukemia (T-PLL) [12]. The success of alemtuzumab in the preclinical studies referenced above has also provided inspiration for a new phase I clinical trial investigating the use of alemtuzumab plus high-dose CTX in the treatment of aggressive non-Hodgkin lymphomas, including DHL and DEL [13].

Previous work has shown significant heterogeneity in CD52 expression by several of the more aggressive mature B-cell lymphomas (e.g. DLBCL, Burkitt lymphoma), with 25% of cases exhibiting negligible CD52 expression by immunohistochemistry [14]. As these earlier immunohistochemical studies predated our current conception of DHL and DEL, the true prevalence of CD52 expression within these newer diagnostic and prognostic categories has remained speculative. To eliminate this knowledge gap and to provide decision support for clinical trial enrollment, we chose to investigate the frequency, intensity and uniformity of CD52 expression within a large collection of DHL and DEL cases. Our results indicate that CD52 is expressed by a significant subset of these aggressive mature B-cell lymphomas, including those from patients with high-risk features and relapsed/refractory disease. CD52 expression status was not correlated with diagnostically relevant pathologic features, necessitating its evaluation on a case-by-case basis for all patients being considered for clinical trial enrollment.

## Methods

DHL and DEL (non-DHL; including both DLBCL and HGBCL, NOS with double-expressor phenotype) surgical and cytology cases were obtained from the files of the Department of Pathology, Brigham and Women's Hospital (BWH), Boston, MA, with institutional internal review board approval. The study was conducted according to the principles expressed in the Declaration of Helsinki. Due to the retrospective nature of the study with use of archival tissues, the IRB waived the requirement for informed consent. DHL cases were identified primarily from BWH cytogenetics reports (FISH and/or karyotype) dating back to 2010, when targeted testing for *MYC*, *BCL2* and *BCL6* translocations became common at our institution. Alternatively, DHL cases were identified primarily through searches of BWH surgical pathology reports, which began to include data pertinent to double-expressor status (i.e. the percentages of MYC and BCL2 positive cells) following the publication of the 2016 revision of the World Health Organization classification of lymphoid neoplasms [2]. Further investigation was performed in all cases for which unstained slides and/or paraffin-embedded tissue sections or cell block preparations were available for additional testing. In situations where multiple specimens derived from the same patient, a single specimen was selected based on fixation/preparation quality and the amount of lesional tissue remaining. Pathologic diagnoses were established according to the 2017 WHO Classification of Tumours of Haematopoietic and Lymphoid Tissues (revised 4^th^ edition) using a combination of morphologic, immunophenotypic, and cytogenetic/FISH findings [15]. Diagnoses were confirmed by review of the original pathology reports and by re-review of H&E stained sections. Cases for which this review process was insufficient for a confident diagnosis were not included in the final analysis. Relevant clinical information was extracted from the medical records.

Immunohistochemical staining (IHC) of 5 μm thick paraffin-embedded tissue sections and cell block preparations was performed using a modified protocol based on previously described methods [14]. Briefly, slides were pretreated with 0.06% (w/v) trypsin (ICN Biomedicals) at 37°C for 15 minutes, and then reacted with Peroxidase Block (DAKO) for 5 minutes to quench endogenous peroxidase activity. Primary rat anti-human CD52 antibody featuring the same complementary determining regions as alemtuzumab (clone YTH34.5; Serotec, Oxford, United Kingdom) was applied at an optimized dilution of 1:2000 for 1 hour at room temperature. Slides were then washed in 25 mmol/L of Tris-Cl pH 7.6 with 0.05% Tween 20 (BioLegend). Rabbit anti-rat secondary antibody (Invitrogen) was applied at a dilution of 1:50 for 30 minutes, followed by incubation with PowerVision Poly-HRP anti-Rabbit IgG (Leica Biosystems) for 30 minutes. Immunoperoxidase staining was developed using a 3,3′-diaminobenzidine chromogen (DAKO) according to the manufacturer’s instructions. Slides were then counterstained with either methyl green (DHL) or hematoxylin (DEL), rinsed, dehydrated through alcohols and xylene, and coverslipped. The majority of specimens used in this study were fixed in formalin, however several BM specimens were fixed with alternative reagents, such as B-plus fixative, Bouin's solution or Zenker’s acetic acid fixative. All cases were treated identically, with the exception of those fixed in Zenker’s acetic acid fixative, for which the initial trypsinization step was omitted.

**Fig 1:**
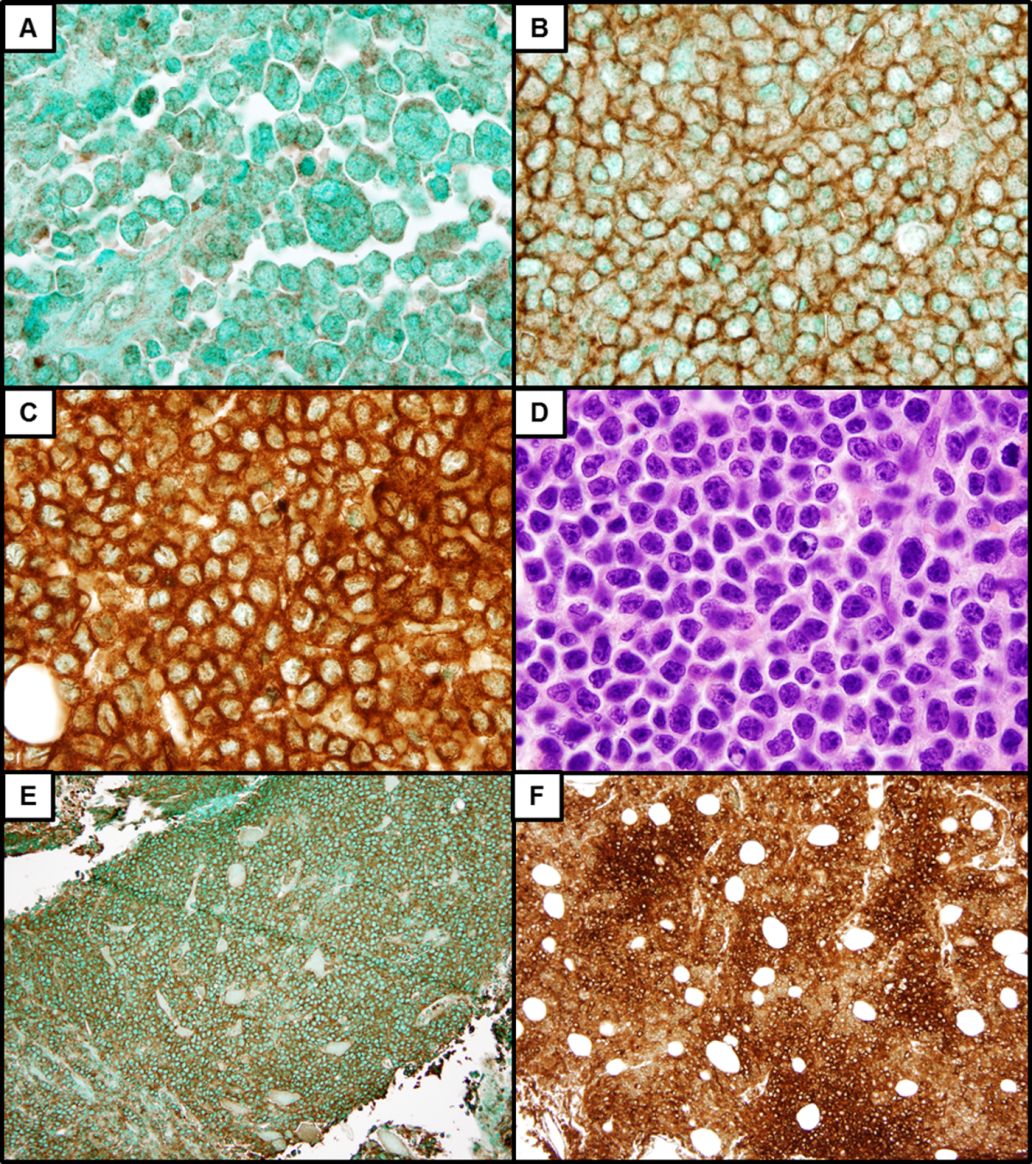
Positive CD52 IHC from representative DHL cases reveals a range of staining intensities: 1+ (A), 2+ (B), and 3+ (C). H&E stain of the strongly CD52-positive DHL case depicted in C (D). Many cases exhibited uniform staining intensity (E), while others showed variable CD52 expression (F).

Reactivity for CD52 was scored independently by two hematopathologists (JWC and OP), with discrepancies resolved at the microscope. Samples were considered positive for CD52 expression if unequivocal positive staining, in a membranous and/or cytoplasmic distribution, was observed in ≥ 50% of the lesional cells within the most well-preserved areas of each specimen. The predominant staining intensity (0, negative; 1+, weak; 2+, intermediate; 3+, strong) and level of heterogeneity (uniform vs. variable) were also assessed on a case-by-case basis (Fig 1). Several cases exhibited non-specific nuclear staining, which was disregarded in the final analysis. Formalin-fixed paraffin-embedded reactive tonsil was used as positive control tissue. In cases that were negative for CD52 expression, separate populations of CD52-positive lymphocytes served as internal controls.

All statistical analyses were performed in the R statistical and computing environment (R version 3.3, Vienna, Austria). Differences between groups were tested using univariate logistic regression, which collapses to chi-square analysis for two-level predictors. Models were initially assessed that controlled for demographic factors, but inclusion of age and gender were not significant covariates, and the most parsimonious models (univariate) were selected. Where noted in Table 1, family-wise false discovery rate adjusted p-values were calculated for the following families of comparisons between DHL and DEL groups: extranodal sites, specimen type, and immunophenotype.

**Table 1:** Comparison of DHL and DEL (non-DHL) cases.

Characteristic	DHL (n = 40)	DEL (non-DHL) (n = 58)*	*P*	*P^*
Age, years, median (range)	68 (range 21–88)	65 (range 30–92)	0.896	
Gender, male	21 (53%)	37 (64%)	0.265	
IPI at diagnosis, average (range)	3.4 (range 1–5)	2.3 (range 0–5)	<0.001	
LDH, U/L, median (range)	677 (range 107–6411)	238 (118–11743)	0.087	
Stage, average (range)	3.9 (range III-IV)	3.3 (range I-IV)	0.008	
Extranodal sites, average (range)	2.1 (range 0–5)	1.3 (range 0–5)	0.003	
Bulky GI disease	9 (23%)	10 (17%)	0.561	0.561
Bone marrow	15 (38%)	4 (7%)	<0.001	0.001
CNS	7 (18%)	8 (14%)	0.490	0.561
Remission achieved	16 (40%)	28 (48%)	0.377	
Primary refractory status	13 (33%)	17 (29%)	0.514	
Prior chemotherapy	3 (8%)	6 (10%)	0.633	
Transformed disease	14 (35%)	8 (14%)	0.016	
Tissue size, mm^2^, median (range)	52 (3–432 mm^2^)	55 (2–432 mm^2^)	0.841	
Specimen type				
Bone marrow	7 (18%)	1 (2%)	0.022	0.022
Other tissue	29 (73%)	57 (98%)	0.004	0.012
Cytology	4 (10%)	0 (0%)	0.014	0.021
Immunophenotype				
CD20, number positive	36 (90%)	56 (97%)	0.184	
MYC, %, median (range)	85 (25–100%)	60 (40–95%)	0.004	0.012
BCL2, %, median (range)	100 (0–100%)	98 (55–100%)	0.850	0.850
Ki67, %, median (range)	75 (20–95%)	85 (35–100%)	0.012	0.018
CD52, number positive	30 (75%)	44 (76%)	0.920	
Cytogenetics				
*MYC* translocation	40 (100%)	4 (7%)	<0.001	
*BCL2* translocation	30 (75%)	NA/ND		
*BCL6* translocation	15 (38%)	NA/ND		
Morphology				
DLCBL	22 (55%)	51 (88%)	NA	
DLBCL/BL	7 (18%)	6 (10%)	NA	
Blastoid	11 (28%)	1 (2%)	NA	

NA = not applicable; ND = not determined; NOS = not otherwise specified; IPI = International Prognostic Index; GI = gastrointestinal; CNS = central nervous system; LDH = lactate dehydrogenase; DLBCL = diffuse large B-cell lymphoma; BL = Burkitt lymphoma

*MYC-translocated DEL cases were shown to be negative for both *BCL2* and *BCL6* translocations;

*P^ =* Family-wise FDR adjusted *p*-values

## Results

In total, our study included 40 cases of DHL (25 with *MYC* and *BCL2* rearrangements, 10 with *MYC* and *BCL6* rearrangements, and 5 'triple-hit lymphomas' with *MYC*, *BCL2* and *BCL6* rearrangements) and 58 cases of DEL (non-DHL), including 51 cases of DLBCL, NOS (17 of Germinal Center B cell-like [GCB] origin, 34 of non-Germinal Center B cell-like [non-GCB] origin, by Hans criteria [16]) and 7 cases of HGBCL, NOS. Comparison of our DHL and DEL cases by clinical characteristics revealed similar age and gender distributions (Table 1). At the time of diagnosis, patients with DHL were associated with a trend towards higher LDH levels [P = 0.087], more advanced Ann Arbor staging [P = 0.008], involvement of greater numbers of extranodal sites [P = 0.003], and higher overall International Prognostic Index (IPI) scores than patients with DEL [P = <0.001]. Patients with DHL were also significantly more likely to have BM involvement than patients with DEL [P = <0.001], while the frequencies of central nervous system (CNS) involvement and bulky gastrointestinal (GI) disease were similar. There were no significant differences between the proportions of DHL and DEL patients achieving remission or acquiring primary refractory status, respectively. As most cases included in this study were used to establish an initial diagnosis, relatively few DHL or DEL patients had received aggressive chemotherapy prior to specimen acquisition.

Fourteen DHL cases represented transformations from follicular lymphoma (FL), while 8 DEL cases corresponded to transformations from either FL (2) or other low-grade non-Hodgkin B-cell lymphomas (3 from lymphoplasmacytic lymphoma, 2 from CLL, and 1 from marginal zone lymphoma) (Table 1). Consistent with their increased predilection for BM involvement, BM biopsies constituted a greater proportion of DHL specimens than DEL specimens [P = 0.022], however the DHL and DEL case groups examined in this study were otherwise similar in terms of tissue origin and sample size. Immunophenotypic comparison showed that the majority of our DHL and DEL cases were positive for the B-cell marker CD20. BCL2, which by definition was present at high levels (i.e. ≥ 50%) in all cases of DEL, was also detected at high levels in most cases of DHL. MYC expression, however, was slightly higher in DHL cases than in DEL cases, despite the requirement of MYC expression in the latter [P = 0.004]. The proliferative marker, Ki67, was significantly elevated in both DHL and DEL. *MYC* gene translocations were present by definition in all DHL cases, and were also present in 4 cases of DLBCL, NOS with double-expressor phenotype.

**Fig 2:**
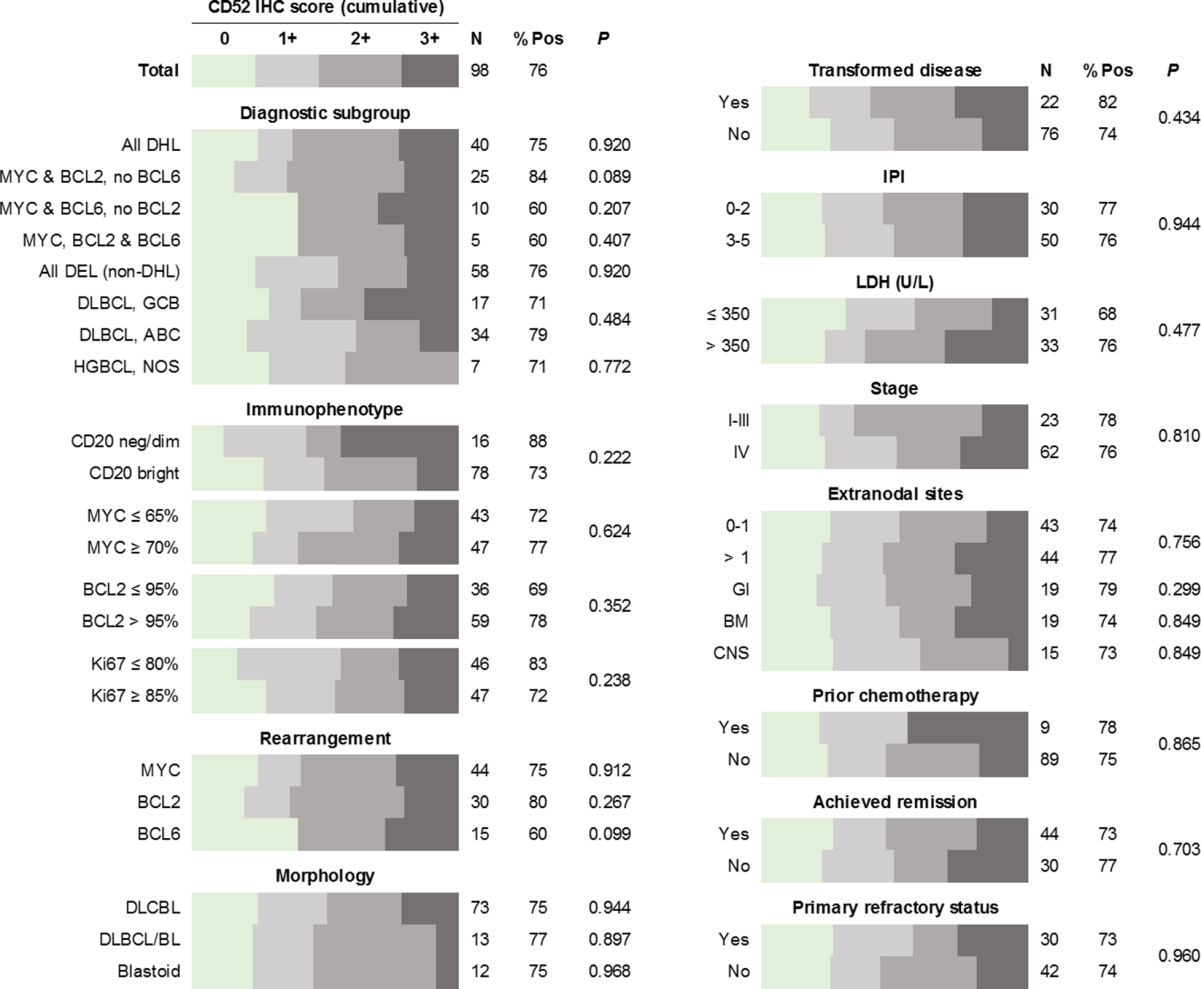
Cumulative bar graphs indicating the relative proportions of DHL and DEL cases with specific CD52 IHC scores (from 0 to 3+), separated into clinical and pathological subgroups.

The results of CD52 IHC show little dependence on predefined clinical characteristics or pathological features (Fig 2). While CD52 expression was highly variable from case to case, convincing cytoplasmic and/or membranous expression was observed in the majority of both DHL (75%) and DEL (75%), at levels consistent with prior studies of aggressive mature B-cell lymphomas[14]. Between the DHL and DEL case groups, there was no significant difference in CD52 staining intensity (scored from 0–3), however DHL cases were significantly more likely to show uniform as opposed to variable staining [P = 0.003]; a finding which held true even after accounting for variations in tissue size. CD52 expression was not affected by LDH level, Ann Arbor stage, extent of extranodal involvement or overall IPI score. The frequency and intensity of CD52 expression in patients with BM disease was similar to that of the entire study group, and the same was true for BM biopsy specimens. Preservation of CD52 expression was also noted in patients with primary refractory disease and/or failure to achieve remission.

Notably, patients previously treated with rituximab-based chemotherapy regimens frequently had CD52-positive disease (78%), including all 6 cases that were negative for CD20 expression. The results of additional immunohistochemical studies (BCL2, MYC, Ki67) showed no impact on CD52 expression status, and tumor morphology (blastoid vs. DLBCL vs. DLBCL/BL) was similarly non-predictive. Within the DHL group, the presence or absence of *BCL2* and/or *BCL6* translocations showed no significant influence over CD52 expression, although there was a slight trend towards increased CD52 expression in the absence of *BCL6* translocation [P = 0.099]. Among cases of DLBCL, NOS with double-expressor phenotype, the Hans cell-of-origin classification (GCB vs. non-GCB) showed no apparent effect on CD52 expression levels.

## Discussion

Treatment regimens incorporating the anti-CD20 monoclonal antibody rituximab have been the cornerstone of therapy for aggressive B-cell lymphomas for nearly two decades [17]. Despite the improvement in survival compared with chemotherapy alone, one third of patients with aggressive B-cell lymphomas are not cured by standard rituximab-based therapies [18]. Many of these failures are attributed to DHL and DEL, and such cases are thought to have benefitted little from the addition of rituximab to standard chemotherapy regimens [19–21]. The high frequency of primary refractory disease in patients with DHL is further complicated by the disappointing results of rituximab-based salvage strategies [5, 22]. In this setting, acquired resistance to chemo-immunotherapy represents a significant source of treatment failure. Loss of CD20 expression on the surface of lesional B-cells is perhaps the most significant mechanism of rituximab resistance in aggressive B-cell lymphomas, however, this phenotypic change is not always present and multiple alternative mechanisms are likely to contribute [23–25]. The poor survival in patients with relapsed/refractory DHL highlights the need for novel therapeutic strategies that are able to utilize alternatives to rituximab-based therapy [26]. Alemtuzumab, which targets CD52 expression on the surface of lesional lymphocytes, also has a well-established track record in the treatment of lymphoid malignancies and represents one of the more obvious alternative agents to consider in this setting.

The presence and extent of extranodal involvement remain independent prognostic factors for patients with aggressive B-cell lymphomas [27]. Certain sites of extranodal involvement have a profoundly negative impact on prognosis, including BM [28, 29]. Up to one-quarter of patients with aggressive B-cell lymphomas have concordant BM involvement at the time of initial diagnosis, and this subset of tumors has been associated with adverse molecular characteristics and gene expression signatures [30]. DHL, in particular, has been repeatedly shown to exhibit a higher frequency of BM involvement compared to conventional DLBCL and other lymphoma subtypes [31]. The difficulty in eradicating medullary disease is due in large part to the BM microenvironment, which is capable of promoting chemoresistance through several mechanisms, including the inhibition of antibody-mediated phagocytosis by BM macrophages [6]. While tissue macrophages have the potential to serve as critical effectors of the anti-tumor immune response, including those mediated by therapeutic antibodies, tumor-associated macrophages have also been linked to tumor-promoting inflammatory programs in multiple cancer types [32]. There is now abundant evidence suggesting that the balance between macrophage mediated pro-tumor and anti-tumor activities is modulated by specific chemotherapeutic agents [33].

A remarkable example of such influence comes from the Hemann laboratory at the Koch Institute/MIT, who recently developed a treatment-refractory humanized mouse model of DHL through the B-cell-specific co-expression of *MYC* and *BCL2* in mice reconstituted with human hematopoietic stem cells [34]. This strategy resulted in the development of a disseminated and aggressive human malignancy that effectively recapitulated the pathological and clinical characteristics of DHL. Using this model, alemtuzumab induced a robust therapeutic response in the peripheral blood and spleen of recipient mice, while BM-based disease remained largely refractory to therapy despite preserved antibody binding [7]. The co-administration of alemtuzumab and high-dose CTX, however, resulted in a strikingly synergistic therapeutic effect, leading to the near-complete eradication of medullary disease [7]. The molecular and cellular underpinnings of this synergism were shown to be due to the ability of CTX to inhibit the secretion of PGE2 and induce the secretion of IL8, TNFα, VEGF, and CCL4 by leukemic cells. These alterations in inflammatory mediators resulted in the progressive recruitment of activated BM macrophages with enhanced phagocytic activity [7]. The potential for alemtuzumab plus high-dose CTX to benefit patients with relapsed/refractory DHL, DEL and other aggressive lymphomas with unmet clinical need has since led to the opening of a phase I trial featuring this synergistic antibody/drug combination [13].

As nearly all studies investigating CD52 expression by aggressive B-cell neoplasms were performed prior to the establishment of our current working definitions of DHL and DEL, such studies failed to differentiate these more aggressive lymphomas from conventional DLBCL. Considering the many biological differences between these neoplasms, we believed it necessary to revisit this topic more formally following the recent release of the 2017 WHO Classification of Tumours of Haematopoietic and Lymphoid Tissues (revised 4^th^ edition) [15]. The results of our study indicate that CD52 is expressed by a significant fraction of both DHL and DEL (75% each), making them potentially amenable to alemtuzumab-based therapy in the appropriate clinical setting. Furthermore, we show that CD52 expression is present at similarly high frequency and intensity in patients who were previously exposed to rituximab-based therapies, including those whose disease exhibits absent or markedly diminished CD20 expression (88%), as well as in patients with various high-risk factors, including BM involvement (74%), higher IPI score (76%), and primary refractory status (73%). The lack of an association between CD52 expression and diagnostically relevant pathologic features (e.g. morphology, immunophenotype and cytogenetic findings) precludes their use as surrogate markers of CD52 expression status. Consequently, evaluation of CD52 expression by IHC or an alternative methodology (e.g. flow cytometry) must be applied on a case-by-case basis to guide eligibility for clinical trial enrollment.

Flow cytometric assessment of CD52 is now available in many clinical laboratories, but may not represent a universal replacement for IHC due to technical challenges that can impact the workup of DHL and DEL specimens. Several common features of aggressive B-cell lymphomas, including increased cell size, cell fragility and frequent association with sclerosis and necrosis, have the potential to result in non-diagnostic flow cytometry studies [35]. Furthermore, the judicious allocation of fresh material for flow cytometry is dependent on clinical situation and diagnostic suspicion, whereas IHC can be performed retrospectively on fixed tissue. Evaluation of CD52 mRNA levels might also be considered as an alternative mechanism for assessing CD52 expression status, perhaps as part of a larger Lymph2Cx-type expression assay compatible with archival tissue [36]. However, the inadvertent inclusion of CD52-positive non-malignant cells represents a potentially significant source of background signal, making the results of such testing difficult to interpret. Thus, in the absence of fresh material available for flow cytometry or alternative studies performed on pre-sorted cells, IHC remains the preferred method for determination of CD52 expression status due to its allowance for direct scoring of malignant cells. Regardless of which technique is used, however, we feel it is most desirable to perform this evaluation on a specimen obtained as recently as possible prior to the intended trial enrollment date, as the long-term stability of CD52 expression in aggressive B-cell lymphomas has not been rigorously evaluated. For example, plasma cell myeloma has shown both the acquisition and loss of CD52 expression over time, even in the absence of CD52-directed therapy [37]. Further studies designed to investigate the consistency of CD52 expression throughout the course of disease and treatment are warranted.

## Supporting information

S1 Table Primary study data. (XLSX)
